# Standardized and flexible eight colour flow cytometry panels harmonized between different laboratories to study human NK cell phenotype and function

**DOI:** 10.1038/srep43873

**Published:** 2017-03-10

**Authors:** John P. Veluchamy, María Delso-Vallejo, Nina Kok, Fenna Bohme, Ruth Seggewiss-Bernhardt, Hans J. van der Vliet, Tanja D. de Gruijl, Volker Huppert, Jan Spanholtz

**Affiliations:** 1 Glycostem Therapeutics, Oss, The Netherlands; 2 Department of Medical Oncology, VU University Medical Cancer Center Amsterdam, De Boelelaan 1117, 1081 HV Amsterdam, The Netherlands; 3 Miltenyi Biotec GmbH, Bergisch Gladbach, Germany; 4 Department of Haematology and Medical Oncology, University Hospital of Wuerzburg, Wuerzburg, Germany; 5 Department of Haematology and Medical Oncology, Sozialstiftung Bamberg, Bamberg, Germany

## Abstract

Advancements in multi-colour fluorescence activated cell sorting (FACS) panel warrant harmonized procedures to obtain comparable data between various laboratories. The intensifying clinical exploration of Natural Killer (NK) cell-based immunotherapy demands standardized and harmonized NK cell FACS panels and acquisition protocols. Eight colour FACS panels were designed to study human NK cell phenotype and function within peripheral blood mononuclear cells (PBMC). The panels were designed around fixed backbone markers and channels, covering antigens for non-NK lineage exclusion (CD3, TCRγδ, CD19, CD14, SYTOX^®^ Blue) and NK cell selection (CD45, CD56, CD16), complemented with variable drop-in markers/channels to study NK cell phenotype (NKG2A, NKG2C, NKG2D and KIR2D) or NK cell function and activation (CD25, NKp44 and CD107a). Harmonized FACS set-up and data analysis for three different flow cytometers has been established, leading to highly comparable and reproducible data sets using the same PBMC reference samples (n = 6). Further studies of NK cells in fresh or cryopreserved PBMC samples (n = 12) confirmed that freezing and thawing of PBMC samples did not significantly affect NK phenotype or function. In conclusion, our data demonstrate that cryopreserved PBMC samples analysed by standardized FACS panels and harmonized analysis protocols will generate highly reliable data sets for multi-center clinical trials under validated conditions.

Flow cytometry serves as a powerful analytical platform for rapid measurement, characterization and functional analysis of individual cells within heterogenic cell populations^1^. The ability to simultaneously detect multiple parameters in different cell types, promoted fluorescent activated cell sorting (FACS) analysis as a crucial tool to study the complexity of the immune system^2^. Recent advances in flow cytometry instruments and reagents have increased the possibilities for development of more complex multi-colour FACS panels, resulting in their extended use in research and clinical studies^3^. Multi-colour FACS panels facilitate a deeper understanding of the biology, distribution and interaction of different immune cell types, offering valuable information to more accurately diagnose, monitor and treat various immunological disorders and malignancies^4^,^5^. There is an ever-increasing number of multi-center clinical trials studying cellular therapy approaches. Thus, immune monitoring of patients should be eased using harmonized multi-colour FACS panels to yield reliable and reproducible data. However, despite the routine use of multi-colour FACS panels in such trials, limitations of implementing standardized methodologies and data analysis protocols have led to a high degree of variation, severely limiting data interpretation from different centers^6^,^7^.

Extensive work done by several groups has identified the main issues that need to be carefully considered when developing multi-colour flow cytometry panels for harmonized use^8^,^9^,^10^, which involve sample type, sample handling, panel design, selection of reagents, instrument set-up, and data analysis. They have also created a series of guidelines recommended to harmonize those processes. Briefly, the design of optimal multi-colour FACS panels requires careful selection of the most appropriate fluorochrome-conjugated antibodies to identify and characterize rare cell populations^11^. Prior to sample acquisition, it is crucial to optimize instrument settings, involving fine-tuning of the light scatters and photomultiplier tube (PMT) voltages for each detector, followed by accurate compensation for spectral overlap of all fluorochromes used. Furthermore, standard operating procedures (SOPs) for sample preparation, staining, acquisition, gating strategy and data analysis methods are essential to reduce data variability of multi-center FACS monitoring. Most of the available multi-colour FACS panels for immune subset analysis are designed for general characterization of major leukocyte populations^2^,^3^,^12^. There is an obvious need for similarly standardized and harmonized multi-colour FACS panels for specific subsets such as for instance natural killer (NK) cells. In particular, their increased use in cellular therapy approaches, as they are perceived as a safer option for targeted anti-cancer therapy than T cells^13^, calls for the development of NK specific polychromatic FACS panels.

NK cells are innate lymphocytes mediating cytotoxic responses against virally infected or tumour cells. The vast majority of peripheral blood NK cells are CD56+CD16+ effector cells and only a small subset represents CD56+CD16− regulatory cells^14^. Their function is tightly regulated by a delicate balance between inhibitory and activating receptors, among which CD16, a low affinity receptor for the Fc fragment of IgG_1_, enables NK cell mediated cytotoxicity of IgG_1_-coated cells, a phenomenon known as antibody dependent cellular cytotoxicity (ADCC)^15^. Although NK cells are involved in the outcome of important clinical interventions that are frequently monitored by multi-colour flow cytometry, such as transplantation^16^,^17^,^18^ or immunotherapy^19^, the existing multi-colour FACS panels for NK cell analysis are either restricted to detect antigens associated with malignant transformation^12^ or if they include an extended immunophenotyping panel, their standardized implementation is limited by the fact that measurements have not been validated through harmonized procedures across multiple centers^20^.

In this article, we describe the design and harmonization of two eight colour NK FACS panels, allowing the generation of reproducible similar data sets across multiple centers, highlighting the advantages of using cryopreserved PBMC for phenotypic and functional immune monitoring studies of NK cells^21^,^22^.

## Results

### NK FACS panel establishment based on backbone and drop-in concept

**Figure 1:**
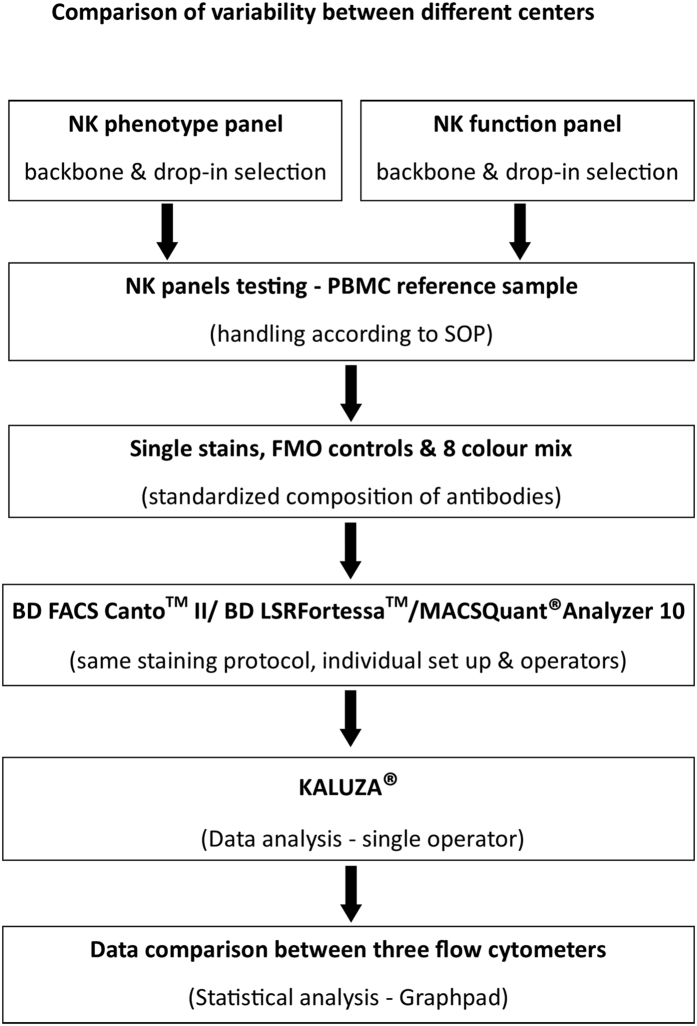
Experimental design to compare variability between flow cytometers. Flow chart of the experimental set up outlining the establishment and verification of the NK cell phenotype and function panels to compare variability between flow cytometers. Following selection of backbone and drop-in antibodies, reference samples were used for optimizing and checking fluorochrome intensities using appropriate single stains and fluorescence minus one (FMO) controls. Further, the data generated from three instruments were analysed using KALUZA^®^ software and results were tabulated using Graphpad Prism for statistical differences for validation using the same reference sample.

To harmonize multicolour flow cytometry analysis for studying NK cell phenotype and function, three independent research centers using different flow cytometers equipped with compatible laser and detector/filter settings (Table 1) tested comparability and reproducibility of obtained data sets between centers. To this end, instrument set-up, sample preparation, acquisition and data analysis were performed independently using standardized protocols, which were commonly agreed on and followed in all three centers as described in the materials and methods section (Fig. 1). Acquisition protocols were set up in each center using single stains, complete mixture of all antibody-fluorochrome combinations and fluorescence minus one (FMO) controls (Supplementary Fig. S1). Compensation matrices of the NK phenotype and function panels were generated for the three flow cytometers and these settings were used through the whole study (Supplementary Tables S1, S2 and S3). FACS panels were designed using a backbone and drop-in concept. Antigens of the backbone specifically discriminated the viable NK cell subset from PBMC, leaving four other detectors available to characterize the specific NK cell phenotype or function.

The backbone of the NK phenotype panel (summarized in Table 2) comprises a combination of CD3, TCRγδ, CD14 and CD19 lineage-specific antibodies (for negative selection of the NK cells), and CD45 and CD56 (for positive selection). In this respect it is important to remark on the extensive overlap between NK cells and γδT cells in terms of the expression of several NK cell receptors^23^; therefore, we included an anti-TCRγδ antibody in the lineage cocktail to avoid misinterpretation of results. Viable CD45+ CD56+ NK cells were gated and CD16 APC, PanKIR2D FITC, NKG2A PE-Vio770, NKG2C PE and NKG2D PerCP-Cy5.5 (drop-ins) were plotted against CD56 to quantify the percentage of NK cells positive for activating (CD16, NKG2C and NKG2D) and inhibitory (KIR2D and NKG2A) receptors (Table 2 and Supplementary Fig. S2). The backbone of the NK cell function panel, was slightly modified to acquire simultaneous information on NK cell, T cell and CD3+CD56+ non-conventional T cell subsets. Here, a combination of CD3 and TCRγδ conjugated to PerCP-Vio700 was used in one of the drop-in channels and removed from the exclusion channel covering CD14, CD19 conjugated to VioBlue and SYTOX^®^ Blue for dead cell exclusion (Table 3). Separately gated NK cells (CD56+/CD3−), T cells (CD3+CD56−) and CD3+CD56+ cells (within the CD45^+^ gate) were then analysed for degranulation^24^ (by CD107a), ADCC potential (by CD16)^25^, and activation status (by CD25, NKp44)^26^,^27^ (Table 3 and Supplementary Table S3). By developing this standardized and harmonized gating strategy a solid basis was set to check the individually developed acquisition protocols of all three participating centers for inter-center variability.

### Standardized FACS panels and protocols overcome variability between flow cytometers and centers

**Figure 2:**
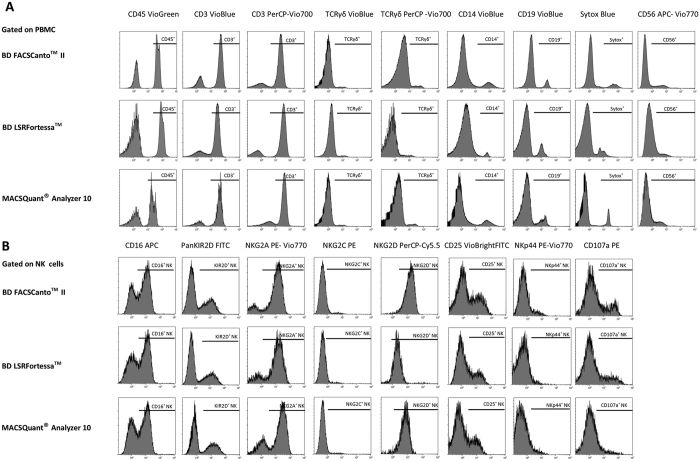
Comparison of FACS panels antibody fluorochrome intensities between three flow cytometers. All antibodies in the NK cell phenotype and function panel were tested for their fluorochrome intensities using the same staining and acquisition protocols on three different flow cytometers. Single stains from backbone antigens were evaluated for their antigen expression levels. PBMC were gated on NK cells for detecting NK receptors and NK functional antigens in the drop-in channels. One representative set of histograms (n = 3) is shown for each marker tested at three different centers using three different flow cytometers. Antigen expression is expressed as percentage of positive cells for backbone antibodies in (**A**) and for NK cell drop ins in (**B**).

**Figure 3:**
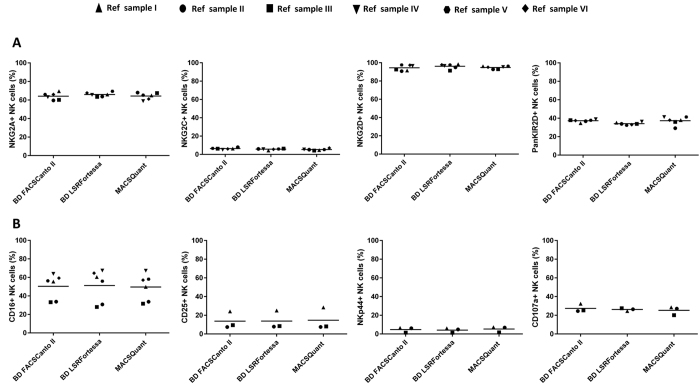
Evaluation of NK cell phenotypes and function marker expression between different flow cytometers. Expression levels for drop-in antigens in the NK cell phenotype (**A**) and function panel (B) were analysed and compared across different centers using the same reference samples (n = 6). NKG2A, NKG2C, NKG2D, KIR2D and CD16 expression in BD LSRFortessa^TM^, BD FACSCanto^TM^ II and MACSQuant^®^ Analyzer 10 were measured for NK phenotype panel (A). Similarly, for NK cell function panel analysis, three time points of reference sample PBMC were stimulated with A431 cells and their percentage of NK cells positive for CD107a, CD25 and NKp44 levels were measured (**B**). Statistical analysis was performed using the non-parametric Kruskal-Wallis test.

The initial selection of antibodies to design NK cell FACS panels, handling of samples and development of a common gating strategy, was used to harmonize data acquisition and analysis between the participating centers. To verify that use of the individual instrument settings and optimized protocols would lead to comparable data sets, experiments were performed in three different centers with different flow cytometers using the same reference PBMC samples (n = 6). First, single-stain antigen expression levels, acquired from each center, were compared for the NK cell phenotype and function panel (Fig. 2). Data from all FCS files, collected from individual experiments, were analysed using the harmonized analysis protocol based on a fixed gating strategy. Using a predefined gating strategy, average frequencies (technical replicates divided by 2) for each parameter were compared between the three flow cytometers (BD FACSCanto^TM^ II, BD LSRFortessa^TM^ or MACSQuant^®^ Analyzer 10) at three different sites using a non-parametric Kruskal-Wallis test. With exception of CD14 VioBlue in BD LSRFortessa^TM^ (Reference sample III, **p-value = 0.001) and CD19 VioBlue in MACSQuant^®^ Analyzer 10 (Reference sample III, *p-value = 0.03), the rest of the measured parameters was comparable across different centers (Fig. 2 and Table 4). Specifically looking at the variability of more sensitive antigens used for discrimination of NK phenotype, no differences were detected for expression of NKG2A, NKG2C, NKG2D, KIR2D (Fig. 3A) between the three flow cytometers (p value range 0.09–0.13). In order to detect the NK function-associated antigens, PBMC were exposed to target cells (A431) for 4hr and expression of CD107a, CD25, NKp44 and CD16 levels were analysed (Fig. 3B).

Standardized FACS panels, sample handling, individual acquisition protocols for the three different flow cytometers, as well as harmonized gating strategies for the analysis of the same reference PBMC samples, resulted in a dataset without inter-center variability. These findings thus support the use of these protocols and NK cell FACS panels for use in the monitoring of multi-center trials.

### NK cell phenotype and function is highly preserved in cryopreserved peripheral blood samples

**Figure 4:**
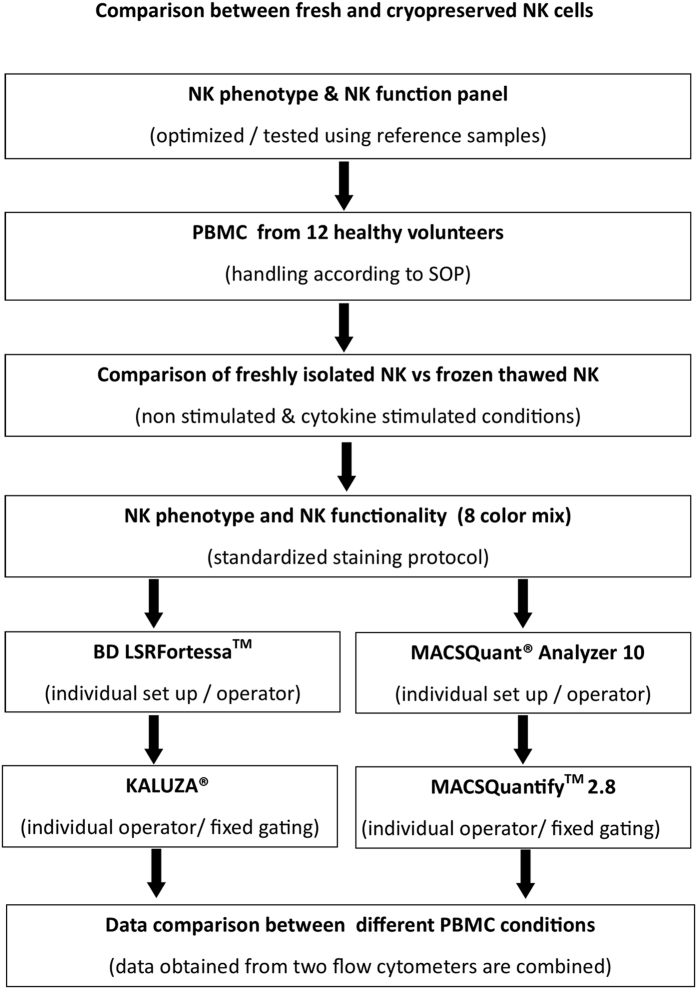
Experimental design to compare different PBMC conditions. The experimental set up describes the work flow to compare NK phenotype and function changes under different PBMC (non-stimulated and stimulated) conditions. PBMC from 12 healthy donors were stained with NK cell phenotype and NK function panel cocktails. Data was acquired for 6 donors using BD LSRFortessa^TM^ and for other 6 donors in MACSQuant^®^ Analyzer 10. Further, FCS files from LSRFortessa^TM^ were analysed on KALUZA^®^ and for MACSQuant^®^ Analyzer 10 in MACSQuantify^TM^ 2.8. Data obtained from both devices were combined and examined for statistical significance between fresh and cryopreserved NK cells.

**Figure 5:**
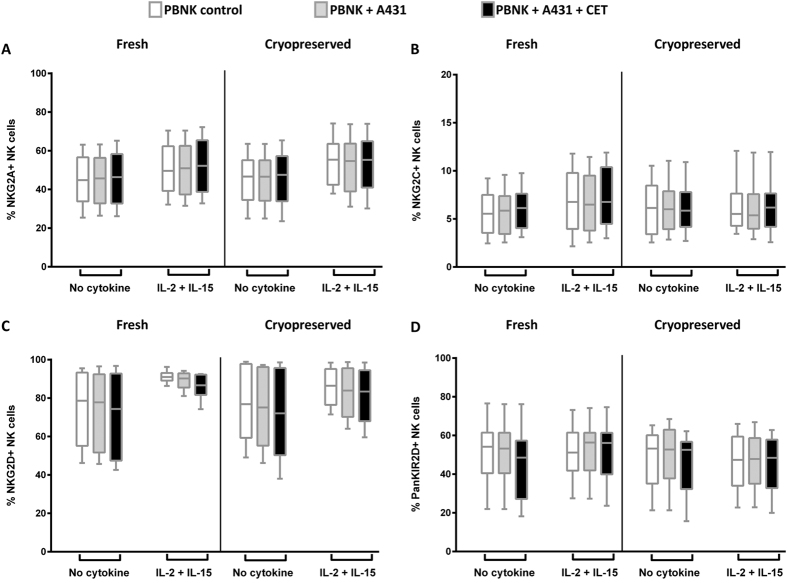
Comparison of NK cell phenotypes between fresh and cryopreserved NK cells. NK cell phenotypes between freshly isolated and cryopreserved thawed PBMC samples were compared. NK cells were either activated or non-activated with cytokines (IL-2+IL-15) and target cells (A431) alone or targets coated with cetuximab (CET). Expression levels of NKG2A (**A**), NKG2C (**B**), NKG2D (**C**), KIR2D (**D**) were compared for the following conditions: i) NK only ii) NK+A431 and iii) NK+A431+CET conditions. NK only conditions are depicted as open rectangles, followed by NK+A431 with grey shades and NK+A431+CET conditions represented as black rectangles. Columns represent data from12 donors, from each donor the mean of triplicate values was used; with bars showing SEM. Data are from independent experiments performed in triplicates from 12 PBMC donors (6 donors: BD LSRFortessa +6 donors: MACSQuant). Statistical analysis was performed using the Wilcoxon test.

With these standardized and harmonized protocols and panels, we initiated a study across the participating partners to compare different handling procedures or culture conditions for PBMC samples to identify the most optimal conditions to study NK cell phenotype and function. For this study, PBMC samples from 12 healthy human blood donors were stained with NK cell phenotype and function panel antibody mixes and were analysed using BD LSRFortessa^TM^ (6 donors) at VUmc and MACSQuant^®^ Analyzer 10 (6 donors) at Miltenyi (Fig. 4). Different PBMC conditions, i.e. freshly isolated PBMC versus cryopreserved PBMC, and cytokine stimulated versus not cytokine stimulated, were compared. To test ADCC effector function, NK cells were exposed to A431 (EGFR positive cell line) cells and A431 cells coated with cetuximab (CET). The expression levels of NK cell phenotype markers were comparatively stable following cryopreservation and no significant differences were observed between fresh and cryopreserved NK cell phenotypes. (Fig. 5, supplementary Figs S4 and S5, and Tables 5 and 6). Upon stimulation with IL-2 and IL-15, though NKG2D expression levels increased resulting in a more homogenous expression level in fresh NK cells compared to their cryopreserved counterparts, however the increase was not significant (Fig. 5C and supplementary Fig. S5C).

**Figure 6:**
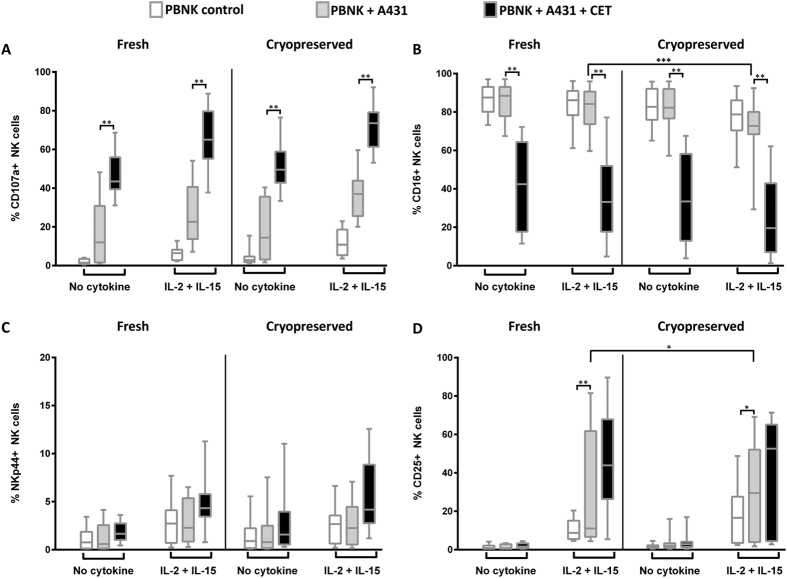
Comparison of NK cell function between fresh and cryopreserved NK cells. NK cell functions between freshly isolated and cryopreserved thawed PBMC samples were compared. NK cells were either activated or non-activated with cytokines (IL-2+IL-15) and target cells (A431) alone or targets coated with cetuximab (CET). Expression levels of CD107a (**A**), CD16 (**B**), NKp44 (**C**), CD25 (**D**) were compared for the following conditions: i) NK only ii) NK+A431 and iii) NK+A431+CET conditions. NK only conditions are depicted as open rectangles, followed by NK+A431 with grey shades and NK+A431+CET conditions represented as black rectangles. Columns represent data from12 donors, from each donor the mean of triplicate values was used; with bars showing SEM. Data are from independent experiments performed in triplicates from 12 PBMC donors (6 donors: BD LSRFortessa+6 donors: MACSQuant). Statistical analysis was performed using the Wilcoxon test.

NK cell activation and function was studied by exposure of NK cells to A431 cells in the presence or absence of CET. The NK function FACS panel was used to detect NK cell degranulation (CD107a levels) and ADCC mediated cytotoxicity comparing differences in CD107a and CD16 cell surface levels between PBMC+A431+CET and PBMC+A431 conditions. Results show that neither the overall CD107a expression levels upon exposure to A431 target cells or A431 target cells coated with cetuximab, nor the increase in CD107a expression in the cetuximab condition were affected by cryopreservation (Fig. 6A and Supplementary Figs S6A and S7A and Tables 5 and 6). Total NK cell CD16 expression levels (Fig. 6B and Supplementary Figs S6B and S7B and Tables 5 and 6) and CD16 expression levels in the CD56dim CD16+NK cell subset (Supplementary Fig S8 and Tables 5 and 6) were significantly reduced (***p-value = 0.0010) when cryopreserved samples were exposed to A431 alone. When comparing CD16 expression levels in PBNK exposed to A431 and CET, no significant differences were noted between fresh and cryopreserved samples. Further, no significant changes in NK cell expression of the activation marker NKp44 could be detected between fresh and cryopreserved nor between non-activated and cytokine activated NK cells27 (Fig. 6C). Upon exposure to A431 cells, freshly isolated NK cells that were stimulated with cytokines expressed significantly higher (*p-value = 0.04) levels of CD25 when compared to NK cells from cryopreserved samples (Fig. 6D and Supplementary Figs S6D and S7D and Tables 5 and 6).

## Discussion

Recent advances in multiparametric flow cytometry offer new and exciting opportunities for the in-depth characterization of immune cell subsets in research, diagnosis and treatment^28^. However, insufficient standardization in sample handling, multi-colour panel design, and data analysis often hinders data interpretation in longitudinal studies performed by different laboratories. With this study, we aimed to develop harmonized FACS panels for the phenotypic and functional assessment of NK cells. Standardized methods for instrument set-up, sample preparation, gating strategies, and data analysis have been developed, including the testing of the best suitable antibody-fluorochrome combinations, compatible with the optical configuration of three different flow cytometers.

The FACS panels were designed with a backbone concept, using lineage antigens and a live/dead dye to effectively exclude the non-NK leukocyte populations and dead cells. By allocating the red laser exclusively for CD56 and CD16 detection, the measurement of an array of additional key NK cell antigens from PBMC was facilitated in panels designed to assess either NK phenotype or functions^29^. Moreover, by separating T and NK cell populations in the NK cell function panel, a precise identification and comparison of NK, T and non-conventional T cells (CD3+CD56+) frequencies and their phenotype and functions could be correlated from a single antibody mix. To enumerate NK cells, T cells and non-conventional T cell subset, we made use of a volumetric flow cytometric counting method or, alternatively, counting beads. The availability of three lasers with the flexibility to use eight colours avoided complexities involving fluorochrome spill over^30^. The TCRγδ antibody was included with the aim of simplifying the identification of NK cell populations from CD3dim or negative subsets under diseased conditions and in αβ-T cell depleted grafts. Moreover, several receptors originally identified in NK cells, are also expressed in γδT cells, such as NKG2D, NKp44, NKp30 or DNAM-1^23^, and regulate in great part the cytotoxic responses that also γδT cells mediate against tumors^31^. Both NK panels had a built in dead cell marker which served as an internal control ensuring the quality and viability of PBMC, besides offering investigators to perform simultaneous cell counting on PBMC samples.

The inclusion of CD16 in the panel enables users to gate specifically on the CD56dimCD16+ and CD56brightCD16− subsets in parallel^14^. Further, CD45+CD3−CD56− cells can also be studied. This series of options allows users to further study changes in NK cell subsets in e.g. chronic viral infections including human immunodeficiency virus-1 and human cytomegalovirus infections, where NK cell subsets are redistributed with expansion of CD56 negative cells but also make this panel useful in conditions where there is either an increase of CD56+CD16− cells or a decrease in CD56 expression levels^32^. Furthermore, changes in CD16 and CD107a cell surface levels can be used to determine the occurrence of natural killing and ADCC killing of target cells. Generally, after NK cell activation upon target cell encounter, CD16 surface levels are reduced and CD107a appears on the cell surface as a consequence of the release of the content of cytotoxic granules. Binding of CD16 to IgG_1_ coated targets enhances NK cell activation and subsequent cytotoxic responses which is reflected by a more pronounced reduction in CD16 surface levels and increased levels of CD107a on the cell surface; both changes have been well reported in the literature^33^ and also specifically in the presence of e.g. the EGFR-binding monoclonal antibody cetuximab^34^. The role of NK cells in eliminating metastasizing cancer cells is well acknowledged in the field of cancer immunotherapy, resulting in an ever increasing number of clinical trials exploring their therapeutic potential^35^. Over the past decade, the development of several therapeutic IgG_1_ monoclonal antibodies (mAbs) targeting different types of cancer^36^, and the recent demonstration of their clinical efficacy, has enhanced the interest in better understanding their mode of action^37^. Information on NK CD16 levels is critical for clinical trials monitoring efficacy of therapeutic ADCC mAbs. Baseline and post mAb treated PBMC samples can be compared for NK CD16 levels, and this could indicate if NK cells were able to recognize and bind therapeutic mAbs, as an indirect measure of ADCC. In addition, activation status of NK cells can be monitored measuring CD25 and NKp44 levels as included in our NK function FACS panel. The analysis of CD25 and NKp44 expression is useful for instance, to check whether NK cells are pre-activated or sensitized in peripheral blood by exposure to targets or soluble NK activating ligands^38^,^39^,^40^. Furthermore, NKp44 levels can also be useful for follow up in patients who had adoptive transfer of pre-activated NK cells (Dolstra *et al*., manuscript submitted).

The NK cell phenotype panel offers information on major NK cell activating and inhibitory receptors NKG2A, NKG2C, NKG2D and KIR2D alleles, which primarily regulate NK cell activity. In different autoimmune diseases as well as in several cancer types, down regulation of NK cell receptors such as CD16, NKp46, NKp30, NKp44 or KLRB1 and a reduction in NK cell number and functional activity are quite common occurences^41^,^42^,^43^. Though these alterations are not necessarily restricted to NK cells, they underscore the relevance of monitoring immune cell functions in these patients. Our NK cell phenotyping panel will prove useful in monitoring NK cells receptor changes that may affect their ability to lyse tumours.

The most interesting and well known aspect of NK cells is their ability to lyse target cells via the release of cytotoxic granules, or through Fas/FasL and TRAIL receptor interactions^44^,^45^. The flexible design of our panels provides a unique opportunity to measure different NK killing mechanisms, e.g. IFNγ, granzyme B and perforin, by modifying drop-in channel combinations. Similarly, natural cytotoxicity receptors (NKp46, NKp44, NKp30), DNAM-1 and other inhibitory/activating receptors can be analysed, suiting the user’s needs.

We compared fresh and cryopreserved PBMC from healthy volunteers in BD LSRFortessa^TM^ and MACSQuant^®^ Analyzer 10. Though a small decrease in NK cell percentages was observed within living CD45+ populations post cryopreservation, it was minor allowing the acquisition of sufficient events to measure NK cell immune functions. Overall cryopreservation did not have a significant effect on the NK cell phenotype in both non-activated and cytokine activated PBMC samples. On the other hand, the decrease in the NK cell marker CD16 was more prominent in A431 stimulated cryopreserved NK cells. Importantly however, in the ADCC condition (NK+A431+CET), the decrease in CD16 expression levels was comparable between fresh and cryopreserved NK cells. Our study also indicated that there is no need for IL-2 and IL-15 cytokine stimulation preceding *in vitro* NK cytotoxicity experiments in this setting, as the influence of these cytokines was restricted to increase in CD25 expression on NK cells, with significantly higher CD25 levels in A431 stimulated fresh NK cells, but did not translate into significantly higher degranulation. In general, NK cell cytotoxicity assays are performed using fresh specimens, whereas our results here support the feasibility to use cryopreserved NK cells for this type of assays. The use of cryopreserved PBMC further limits the drawbacks involved in real time analysis of fresh samples over different time points, thus reducing instrument variability and allows generating more reproducible data sets in longitudinal analysis.

Multicenter trials should aim for harmonized panels and reproducible data generation, but have faced challenges in this respect due to variability in flow cytometers and antibody-fluorochrome conjugates resulting in inconsistent data sets^46^,^47^,^48^. With our panel design, uniform gating strategy and implementation of standardized procedures, we were able to obtain reproducible data in three different centers, thereby overcoming inter-laboratory variability issues. Further, differences related to instrument set-up or operators, did not significantly influence the data set. The unique ability to obtain highly reproducible data between three flow cytometers independent of operators and machines, offers an ideal opportunity to use these FACS panels in multicenter trials for monitoring of clinical specimens. The generation of comparable results between different centers could further help in designing cost effective clinical trials by reducing shipment costs effectively.

## Material and Methods

### PBMC isolation and activation

Whole blood samples were collected from healthy donors after obtaining informed consent in accordance with the “Code for Proper Use of Human Tissues” as formulated by the Dutch Federation of Medical Scientific Organizations (www.fmwv.nl)^49^. PBMC were isolated from whole blood using Lymphoprep™ (STEMCELL Technologies, Vancouver, Canada) density gradient centrifugation. PBMC were incubated at a concentration of 5 × 10^6^/ml in 6 well plates in Roswell Park Memorial Institute (RPMI) medium (Sigma Aldrich, Zwijndrecht, The Netherlands) containing 2% human serum albumin (HSA) overnight at 37 °C, 95% humidity, 5% CO_2_ atmosphere. PBMC viability, numbers and NK cell content were counted volumetrically by FACS using a cocktail of 7-AAD (1:200) (Sigma Aldrich, Zwijndrecht, The Netherlands), CD3 VioBlue (1:11), CD56 APC-Vio 770 (1:11), CD16 APC (1:11), CD45 VioGreen (1:11), and CD25 VioBrightFITC (1:11) antibodies from Miltenyi Biotec GmbH. PBMC viability (7AAD− CD45+) and NK cell (7AAD− CD45+ CD3− CD56+) and NK CD16 (7AAD− CD3− CD56+ CD16+) percentages measured before and after overnight incubation were 7AAD− CD45 + (lymphocytes): 91% ± 4% versus 84 ± 2%, %; 7AAD− CD45+ CD3− CD56+ (NK cells): 7.1% ± 3% versus 6.8% ± 6% and CD3− CD56+CD16+ (CD16 positive cells within NK cells): 89% ± 3% & 82% ± 9%.

### Cryopreservation and thawing of PBMC

PBMC were suspended at 1 × 10^7^ viable cells/ml in 500 μl human serum albumin (HSA) per tube and 500 μl of freezing medium containing 14% Dimethyl Sulfoxide (DMSO), (Sigma Aldrich, Zwijndrecht, The Netherlands) in Roswell Memorial Park Institute RPMI medium (Sigma Aldrich, Zwijndrecht, The Netherlands) was added to the cells. Isolated PBMC were frozen at a final concentration of 7% DMSO in pre-cooled Nalgene^®^ Mr. Frosty freezing containers (Thermo Scientific, Landsmeer, The Netherlands) overnight at −80 °C and transferred after 24 h to liquid nitrogen for longer storage. To thaw PBMC, cryovials were transferred from liquid nitrogen into a pre-warmed 37 °C water bath. Cells were thawed and washed twice in 500 μl of PBS + 0.5% HSA to remove toxic DMSO and suspended in RPMI medium containing 2% HSA for further studies.

### Sample handling

In the first phase of the study, peripheral blood mononuclear cells (PBMC) from one healthy donor were collected at different time points as reference samples (n = 6) to establish harmonized data acquisition and analysis between three participating centers. All reference samples were isolated and cryopreserved at one center and shipped to other participating centers (n = 3). The cryopreserved samples were thawed and analysed at least one month after PBMC collection at all participating centers. At all centers same 3 time points of reference samples were used to optimize the instrument settings, staining protocols and panels. An additional 3 time points of the same reference sample were stained with the NK cell phenotype panel antibody mix to show the obtention of reproducible data sets across the 3 centers.

In the second phase of the study in which the NK cell phenotype and function was compared between fresh and cryopreserved NK samples, PBMC were collected from different healthy donors (n = 12; 6 samples were processed at Miltenyi Biotech and analysed using a MACSQuant^®^ Analyzer 10 device, and other 6 were processed at VU medisch centrum and analysed using BD LSRFortessa^TM^. One fraction of the isolated PBMC was cryopreserved and another fraction was used directly for NK cell analysis. Further, for testing differences in the NK cell phenotype and function between non-activated and cytokine activated samples, both fresh and cryopreserved samples were split into 2 fractions. One fraction was activated overnight with 1000U/ml of IL-2 (Proleukin^®^; Chiron, München, Germany) and 10ng/ml of IL-15 (CellGenix GmbH, Freiburg, Germany), while the other fraction was incubated without cytokines.

### Cell line

The A431 (epidermoid carcinoma) cell line was obtained from ATCC and cultured in Dulbecco’s modified medium (DMEM; Invitrogen, Carlsbad CA, USA) containing 100 U/ml penicillin, 100 μg/ml streptomycin and 10% fetal calf serum (FCS; Integro, Zaandam, The Netherlands). Cells were passaged every 5 days and early-passage cells (passage numbers between 20–25) were used for the experiments. Cells were maintained in a 37 °C, 95% humidity, 5% CO_2_ atmosphere.

### Anti - EGFR monoclonal antibody

The anti-EGFR monoclonal antibody cetuximab (Erbitux^®^, Merck, Darmstadt, Germany) was purchased from the VU University Medical Center pharmacy for NK cell ADCC experiments.

### Flow cytometers and instrument settings

Experiments were performed with three different flow cytometers: BD LSRFortessa^TM^ X-20 (Becton Dickinson (BD) B.V, Breda, The Netherlands), BD FACSCanto^TM^ II (Becton Dickinson B.V, Breda, The Netherlands) and MACSQuant^®^ Analyzer 10 (Miltenyi Biotec GmbH, Bergisch Gladbach, Germany). All devices were equipped with 3 solid state lasers (Violet, Blue and Red). Comparable band pass and long pass filters enabled the use of same fluorochrome-conjugated antibodies between the three machines (Table 1). The chosen fluorochromes were distributed accordingly; two light scatter parameters and 8 fluorescence detectors: Violet laser excited VioGreen and VioBlue fluorochromes, Blue laser for forward and side scatter, besides detecting signals from FITC, VioBrightFITC, PE, PerCP-Cy5.5, PerCP-Vio700 and PE-Vio770 channels and red laser for APC and APC-Vio770. Daily maintenance and initial photomultiplier tube (PMT) voltage determination were performed using recommended cytometer tracking and set-up beads (CS&T) (BD biosciences, Breda, The Netherlands) for BD instruments, and MACSQuant^®^ calibration beads (Miltenyi Biotec GmbH, Bergisch-Gladbach, Germany) for MACSQuant^®^ Analyzer 10.

### Antibodies, staining protocol and compensation settings

The antibody clones and fluorochrome combinations were selected based on lab user experience and antibody sensitivity and with the aim of achieving minimal spill over between detectors. Moreover, the antibody - fluorochrome conjugates were chosen in order to attain high sensitivity for measuring dimly expressed antigens besides overcoming sterical hindrance. For staining, the PBMC concentration was adjusted in washing buffer (PBS containing 0.5% bovine serum albumin) to 1 × 10^6^/ml and stained in 96 well U bottom plates (Corning, Amsterdam, The Netherlands). PBMC were incubated with relevant antibody mixes in a final premix volume of 30 μl for 10 minutes at 4 °C in the dark. After incubation cells were washed once with 170 μl of washing buffer per well and centrifuged at 300 × g for 5 min, supernatants were discarded and cell pellets suspended in 200 μl of washing buffer until measurement. Samples were measured within 1 hour.

The antibody mix for the NK phenotype panel consisted of CD45 VioGreen, CD3 VioBlue, TCRγδ VioBlue, CD14 VioBlue, CD19 VioBlue and SYTOX^®^ Blue for live NK cell discrimination and NK cell phenotypic antigens PanKIR2D FITC, NKG2A PE, NKG2C PE-Vio770 and NKG2D PerCP-Cy5.5. Similarly, the antibody mix for the NK function panel consisted of CD45 VioGreen, CD14 VioBlue, CD19 VioBlue and SYTOX^®^ Blue, together with CD3 PerCP-Vio700 and TCRγδ PerCP-Vio700 to identify NK cells, with inclusion of NK function antigens CD25 VioBrightFITC, CD107a PE, NKp44 PE-Vio770. Most of the antibodies were supplied by Miltenyi Biotec except NKG2D PerCP-Cy5.5 (Biolegend, Fell, Germany) and dead cell stain SYTOX^®^ Blue (Thermo Fisher Scientific, Berlin, Germany). Manufacturer recommended dilutions of the antibodies were first checked and finally used. Details on antibody clones and dilutions used in the NK phenotyping and NK function panel are listed in Tables 2 and 3.

Due to the absence or low expression of some antigens (CD25, NKp44 or CD107a) on NK cells in unstimulated PBMC, we used Ig-capture beads (MACS^®^ Comp Bead anti-mouse Igκ and MACS^®^ Comp Bead anti-human Igκ, Miltenyi Biotec GmbH, Bergisch-Gladbach, Germany) to establish initial fluorescence compensation matrices using the automatic compensation feature of each instrument. Further, unstained PBMC and single staining of all antibodies was acquired to assess the compensation matrices obtained with the Ig-capture beads. In addition to this, FMO controls were performed for drop-in antigens (CD25 VioBrightFITC, NKp44 PE-Vio770 and CD107a PE) in the NK function panel (Supplementary Fig. 1). Compensation matrices were calculated using automated settings from BD FACS DIVA^TM^ software for BD FACSCanto^TM^ II and BD LSRFortessa^TM^, and MACSQuantify^TM^ 2.8 software for MACSQuant^®^ Analyzer 10. In situations where instrument compensation was not optimal, adjustments were made based on the single staining controls from PBMC using KALUZA^®^ data analysis software (Beckman Coulter, California, US). Compensation matrices of the NK phenotype and function panels for the three flow cytometers are shown in supplementary Tables S1, S2, and S3.

### NK cell cytotoxicity assays

Activated and non-activated PBMC (containing effector cells) were stimulated with A431 cells (targets) in the presence or absence of 5 μg/ml of cetuximab (CET) in a total volume of 100 μl in 96 well U bottom plates. PBMC counts were adjusted so that NK cell numbers matched with the number of target cells (5 × 10^4^ target and 5 × 10^4^ NK cells from PBMC) at a 1:1 E: T ratio. PBMC without targets and PBMC+CET without targets were included as controls. All treatments and corresponding conditions were performed in triplicate. After 4 h incubation at 37 °C, 95% humidity and 5% CO_2_ atmosphere, cells were pelleted and stained with the NK phenotype (Table 2) or NK function antibody (Table 3) mixes. To assess degranulation by NK cells, anti-CD107a PE (Miltenyi Biotech GmbH, Bergisch Gladbach, Germany) was added at the beginning of the assay.

### Acquisition and data analysis for harmonisation between different flow cytometers

Individual acquisition protocols following standardized instrument settings were set up in three centers for three different flow cytometers using the same reference samples (n = 6) and the same staining antibody mixtures. In the first step, to compare variability between flow cytometers, reference PBMC samples were isolated and stored as described above for testing. Single-stain controls were acquired for antibody - fluorochrome conjugates in the NK phenotype and NK function panel. Further, to detect expression of NK cell receptors and NK cell function-associated antigens and effector molecules, PBMC were exposed to target cells (A431). FCS files acquired from three flow cytometers were analysed in KALUZA^®^. For comparison of different PBMC conditions (fresh versus cryopreserved, no cytokine versus cytokine activated and no CET versus CET stimulated), data acquisition was done using two flow cytometers BD LSRFortessa^TM^ and MACSQuant^®^ Analyzer 10. In this experimental part, the FCS files obtained were analysed by individual laboratories, with KALUZA^®^ software for analysis of data generated from BD LSRFortessa^TM^ and MACSQuantify^TM^ 2.8 software for data generated from MACSQuant^®^ Analyzer 10.

### Gating strategy

Two different gating strategies were defined, one for the NK phenotype and another for the NK function panel following a modified ISHAGE gating strategy^50^. For the NK phenotype panel, cell doublets were excluded by plotting forward side scatter area and height parameters. Next, CD45 was plotted against CD3/TCRγδ/CD14/CD19 (lineage) and SYTOX^®^ Blue to gate on all live and lineage- CD45+ cells. Thus, selected cells were further plotted against CD56, identifying only viable NK cells. This NK cell gate was used to assess the expression levels of CD16, NKG2A, NKG2C, NKG2D and KIR2D antigens (Supplementary Fig. S2). Of note, the gating strategy for the NK cell function panel was essentially the same with one exception, i.e. the inclusion of CD3 and TCRγδ as drop-in antigens, excluding them from the backbone markers. With this panel, we differentiated between three cell populations: NK cells (CD45+CD3−/TCRγδ−CD56+) T cells (CD45+CD3/TCRγδ+CD56−) and non-conventional T cell subsets (CD45+CD3+/TCRγδ+CD56+). The gate on the NK cell population (CD45+CD3−/TCRγδ− CD56+) was used to analyse the expression of CD107a, CD25 and NKp44 (Supplementary Fig. S3).

### Statistical analysis

Statistical analysis was performed using GraphPad Prism software. Differences between centers were determined using Kruskal Wallis test and difference between fresh and cryopreserved were determined using non-parametric Wilcoxon test. P value of <0.05 was considered statistically significant.

### Ethics Statement

The research presented in this paper, all methods and experimental protocols were approved and carried out in accordance with relevant guidelines and regulations of the Committee for Scientific Research of the VU University Medical Center, Cancer Center Amsterdam.

## Additional Information

**How to cite this article:** Veluchamy, J. P. *et al*. Standardized and flexible eight colour flow cytometry panels harmonized between different laboratories to study human NK cell phenotype and function. *Sci. Rep.* **7**, 43873; doi: 10.1038/srep43873 (2017).

**Publisher's note:** Springer Nature remains neutral with regard to jurisdictional claims in published maps and institutional affiliations.

## Supplementary Material

Supplementary Information

## Figures and Tables

**Table 1:** Comparison of instrument settings between three flow cytometers from three centers participated in the study.

Comparison of instrument settings between BD FACS Canto™ II, BD LSRFortessa™ and MACS Quant^®^ Analyzer 10						
Fluorochromes	Filter settings - Band pass & Long pass filters			PMT voltages		
	BD FACS Canto™ II	BD LSR Fortessa™	MACS Quant^®^ Analyzer 10	BD FACS Canto™ II	BD LSR Fortessa™	MACS Quant^®^ Analyzer 10
FSC	488/10	488/10	488/10	319	489	328
SSC	488/10	488/10	488/10	462	266	476
FL1	530/30 & 502LP	530/30 & 505LP	525/50	496	484	417
FL2	585/42 & 556LP	575/26 & 550LP	585/40	435	473	416
FL3	780/60 & 735LP	780/60 & 750LP	750LP	540	549	487
FL4	670LP & 655LP	695/40 & 685LP	655–730	507	681	594
FL5	660/20	670/14 & 655LP	655–730	588	484	522
FL6	780/60 & 735LP	780/60 & 750LP	750LP	474	451	579
FL7	450/50	450/50	450/50	375	423	444
FL8	510/50 & 502LP	525/50 & 505LP	525/50	403	453	560

Three flow cytometers (BD FACS Canto™ II, BD LSR Fortessa™ and MACS Quant^®^ Analyzer 10) with compatible optical configuration were used for NK phenotype and NK function FACS panel design and optimization studies. Forward scatter (FSC), side scatter (SSC) and eight fluorescence emission channels (FL1-FL8) were used. Further, their corresponding band pass and long pass (LP) filter settings were compared and the photomultiplier tube (PMT) voltages obtained are mentioned.

**Table 2:** Antibody specifications - NK phenotype panel.

Laser	Antibody	Fluorochrome	Clone	Titration	Manufacturer	Catalogue No
Violet 405 nm	**CD45**	VioGreen	5B1	1:11	Miltenyi	130-096-906
	**CD3**	VioBlue	BW264/56	1:11	Miltenyi	130-094-363
	**TCRγδ**	VioBlue	11F2	1:11	Miltenyi	130-101-557
	**CD14**	VioBlue	TÜK4	1:11	Miltenyi	130-094-364
	**CD19**	VioBlue	LT19	1:11	Miltenyi	130-098-598
	**Sytox^®^ Blue**	Dead cell marker		1:1000	Life technologies	S11348
Blue 488 nm	PanKIR2D	FITC	NKVFS1	1:11	Miltenyi	130-098-689
	NKG2A	PE-Vio770	REA110	1:11	Miltenyi	130-105-647
	NKG2C	PE	REA205	1:11	Miltenyi	130-103-635
	NKG2D	PerCP-Cy5.5	1D11	1:11	Biolegend	320818
Red 633 nm	**CD16**	APC	VEP13	1:11	Miltenyi	130-091-246
	**CD56**	APC-Vio770	REA196	1:11	Miltenyi	130-100-694

Antibody-fluorochrome conjugates for NK phenotype panel were distributed across Violet, Blue and Red lasers. Backbone antibodies (Bold) were assigned to Violet and Red lasers. VioGreen was used to gate on CD45+ cells and VioBlue in the Violet laser was used as a dump channel to exclude non-NK lymphocytes including dead cells. Red laser was used to gate on viable NK cells, followed by NK phenotype panel drop-in markers (white shades) in the Blue laser. All antibodies except Sytox Blue (1:1000) were used at a dilution of 1:11.

**Table 3:** Antibody specifications - NK function panel.

Laser	Antibody	Fluorochrome	Clone	Titration	Manufacturer	Catalogue No
Violet 405 nm	**CD45**	VioGreen	5B1	1:11	Miltenyi	130-096-906
	**CD14**	VioBlue	TÜK4	1:11	Miltenyi	130-094-364
	**CD19**	VioBlue	LT19	1:11	Miltenyi	130-098-598
	**Sytox^®^ Blue**	Dead cell marker		1:1000	Life technologies	S11348
Blue 488 nm	CD25	VioBrightFITC	4E3	1:11	Miltenyi	130-104-274
	CD107a	PE	H4A3	1:11	Miltenyi	130-095-515
	NKp44	PE-Vio770	2.29	1:11	Miltenyi	130-104-195
	CD3	PerCP-Vio700	BW264/56	1:11	Miltenyi	130-097-582
	TCRγδ	PerCP-Vio700	11F2	1:11	Miltenyi	130-103-784
Red 633 nm	**CD16**	APC	VEP13	1:11	Miltenyi	130-091-246
	**CD56**	APC-Vio770	REA196	1:11	Miltenyi	130-100-694

Antibody-fluorochrome conjugates for NK function panel were distributed across Violet, Blue and Red lasers. Backbone antibodies (Bold) were assigned to Violet and Red lasers. VioGreen was used to gate on CD45+ cells and VioBlue in the Violet laser was used as a dump channel to exclude CD14+, CD19+ and dead cells. Red laser was used to gate on viable NK cells. Blue laser was used to gate on CD3+, TCRγδ+ cells, non-conventional T cell subsets and NK function panel drop-in markers (white shades). All antibodies except Sytox Blue (1:1000) were used at a dilution of 1:11.

**Table 4:** Comparison between instruments of the increment in average cell frequencies obtained after staining with the panels components.

Antibodies	Reference sample I				Reference sample II				Reference sample III			
	Canto	Fortessa	MQ	p- value	Canto	Fortessa	MQ	p- value	Canto	Fortessa	MQ	p- value
CD45 VioGreen	98.3	97.2	98.3	ns	99.5	94.0	98.5	ns	97.0	97.0	99.0	ns
CD3 VioBlue	75.6	73.0	76.7	ns	75.3	73.4	77.5	ns	67.2	69.0	71.7	ns
CD14 VioBlue	1.13	2.5	1.0	ns	1.11	3.7	1.0	ns	1.7	12.92	1.1	**
CD19 VioBlue	16.0	16.4	14.5	ns	7.7	7.7	12.5	ns	4.19	9.76	20.2	*
**Sytox^®^ Blue**	17.0	12.4	18.5	ns	16.2	13.1	14.5	ns	13.6	12.3	19.0	ns
CD56 APC-Vio770	2.9	2.5	2.4	ns	2.2	3.0	2.7	ns	9.29	9.8	8.3	ns
CD3 PerCP-Vio700	76.3	74.0	77.5	ns	74.6	77.5	77.4	ns	68.4	69.7	72.1	ns
TCRγδ PerCP-Vio700	1.9	2.5	2.9	ns	1.7	2.7	3.23	ns	2.6	2.5	2.9	ns
TCRγδ VioBlue	1.6	1.6	2.4	ns	1.7	1.4	2.71	ns	2.5	2.7	2.4	ns
CD56^+^CD16 APC	33.0	28.0	31.6	ns	33.4	30.0	33.7	ns	36.0	34.5	31.5	ns
**CD56^+^NKG2A PE-Vio770**	59.0	63.4	57.5	ns	59.6	63.8	61.0	ns	48.8	51.6	62.9	ns
**CD56^+^NKG2C PE**	6.3	6.2	6.4	ns	7.9	4.9	6.0	ns	11.0	10.7	12.7	ns
**CD56^+^NKG2D PerCP-Cy5.5**	92.6	91.3	92.9	ns	90.8	94.9	92.8	ns	97.8	95.5	93.5	ns
**CD56^+^PanKIR2D FITC**	35.1	33.9	36.0	ns	34.2	33.9	29.2	ns	18.1	19.3	21.4	ns
***CD56^+^CD25 VioBrightFITC***	7.2	7.8	8.0	ns	6.9	7.2	7.4	ns	22.3	23.4	25.2	ns
***CD56^+^NKp44 PE-Vio770***	1.0	0.8	1.2	ns	5.6	4.4	6.6	ns	6.1	6.0	7.5	ns
***CD56^+^CD107a PE***	23.7	24.2	19.5	ns	24.0	25.2	26.3	ns	31.2	28.2	33.8	ns

Three reference samples (Reference sample I–III) were analysed using three different flow cytometers at three different centers by independent operators using an optimized protocol. The performance of the backbone antibodies (white shades) was evaluated using single stain controls for each measured parameter with background subtracted from corresponding negative controls. The drop-in markers for NK phenotype panel (Bold) were analysed, when gated on NK cells (CD45+CD3−CD56+). Background staining in NK phenotype panel drop-in channels were eliminated using appropriate fluorescence minus one (FMO) controls. For the NK function panel drop-in markers; CD107a, CD25 and NKp44, NK cells were stimulated with A431 cells and unstimulated values were subtracted before analysis (Italics). The values shown for each parameter correspond to the average frequency of stained cells from two technical replicates measured in triplicates, following subtraction of appropriate background signal. The statistical comparisons were performed using non-parametric Kruskal-Wallis. Only significant p-values (<0.05) are shown.

**Table 5:** Comparison of NK phenotype and function parameters between non-activated fresh and cryopreserved samples.

Non-activated fresh versus cryopreserved NK samples (Wilcoxon test)			
NK FACS panel drop-ins	NK only	NK+A431	NK+A431+CET
CD56^+^NKG2A PE-Vio770	ns	ns	ns
CD56^+^NKG2C PE	ns	ns	ns
CD56^+^NKG2D PerCP-Cy5.5	ns	ns	ns
CD56^+^PanKIR2D FITC	ns	ns	ns
CD56^+^CD107a PE	ns	ns	ns
CD56^+^CD16 APC	ns	ns	ns
CD56^+^NKp44 PE Vio770	ns	ns	ns
CD56^+^CD25 VioBrightFITC	ns	ns	ns

Non-activated freshly isolated NK cell samples from 12 healthy donors and their corresponding cryopreserved counterparts were stained with the NK phenotype and NK function antibody mix and compared under different stimulation conditions (NK only, NK+A431, NK+A431+CET). All samples were measured in triplicates. Statistical analysis to detect differences between non-activated fresh and cryopreserved samples was performed using the Wilcoxon test. No significant p-values (<0.05) were obtained for the assessed NK phenotype and function panel drop-in parameters. Marker expression level of individual donors are shown in supplementary Figs S4, S5 and S8B.

**Table 6:** Comparison of NK phenotype and function parameters between cytokine activated fresh and cryopreserved samples.

Activated fresh versus cryopreserved NK samples (Wilcoxon test)			
NK FACS panel drop-ins	NK only	NK+A431	NK+A431+CET
CD56^+^NKG2A PE-Vio770	ns	ns	ns
CD56^+^NKG2C PE	ns	ns	ns
CD56^+^NKG2D PerCP-Cy5.5	ns	ns	ns
CD56^+^PanKIR2D FITC	ns	ns	ns
CD56^+^CD107a PE	ns	ns p = 0.06	ns
CD56^+^CD16 APC	ns	***p = 0.0010	ns
CD56^+^NKp44 PE Vio770	ns	ns	ns
CD56^+^CD25 VioBrightFITC	ns	*p = 0.03	ns
CD56^dim^ CD16 APC	ns	***p = 0.0010	ns

Cytokine activated freshly isolated NK cell samples from 12 healthy donors and their corresponding cryopreserved counterparts were stained with the NK phenotype and NK function antibody mix and compared under different stimulation conditions (NK only, NK+A431, NK+A431+CET). All samples were measured in triplicates. Statistical analysis to detect differences between non-activated fresh and cryopreserved samples was performed using the Wilcoxon test. No significant p-values (<0.05) were obtained for the assessed NK phenotype and function panel drop-in parameters. Marker expression level of individual donors are shown in supplementary Figs S6, S7 and S8C.
