# Supplementary material for: Standardized and flexible eight colour flow cytometry panels harmonized between different laboratories to study human NK cell phenotype and function
Source: Sci Rep. 2017 Mar 10;7:43873. doi: 10.1038/srep43873 (PMC5345017; doi:10.1038/srep43873)
Supplement: Supplementary Information [file srep43873-s1.pdf]

Standardized and flexible eight colour flow cytometry panels harmonized between different laboratories to study human NK cell phenotype and function

John P Veluchamy[1,2†], María Delso-Vallejo[3†], Nina Kok[1], Fenna Bohme[1], Ruth Seggewiss-Bernhardt[4,5], Hans J. van der Vliet[2], Tanja D. de Gruijl[2], Volker Huppert[3] and Jan Spanholtz[1*]

[1]Glycostem Therapeutics, Oss, The Netherlands

[2]Department of Medical Oncology, VU University Medical Cancer Center Amsterdam, De Boelelaan 1117, 1081 HV Amsterdam, The Netherlands

[3]Miltenyi Biotec GmbH, Bergisch Gladbach, Germany

[4]Department of Haematology and Medical Oncology, University Hospital of Wuerzburg, Wuerzburg, Germany

[5]Department of Haematology and Medical Oncology, Sozialstiftung Bamberg, Bamberg, Germany

[†] Veluchamy JP and Delso-Vallejo M contributed equally to this paper.

Contact information:

Jan Spanholtz, Glycostem Therapeutics,

Pivot Park RE1134 |Kloosterstraat 9 | 5349 AB Oss, The Netherlands

Email: jan.spanholtz@glycostem.com / Tel: +31-(0) 412 211 001

Keywords: multi-colour FACS panels, harmonization, multi-center trials, NK phenotype panel, NK function panel, cryopreservation.

Supplementary Fig. S1

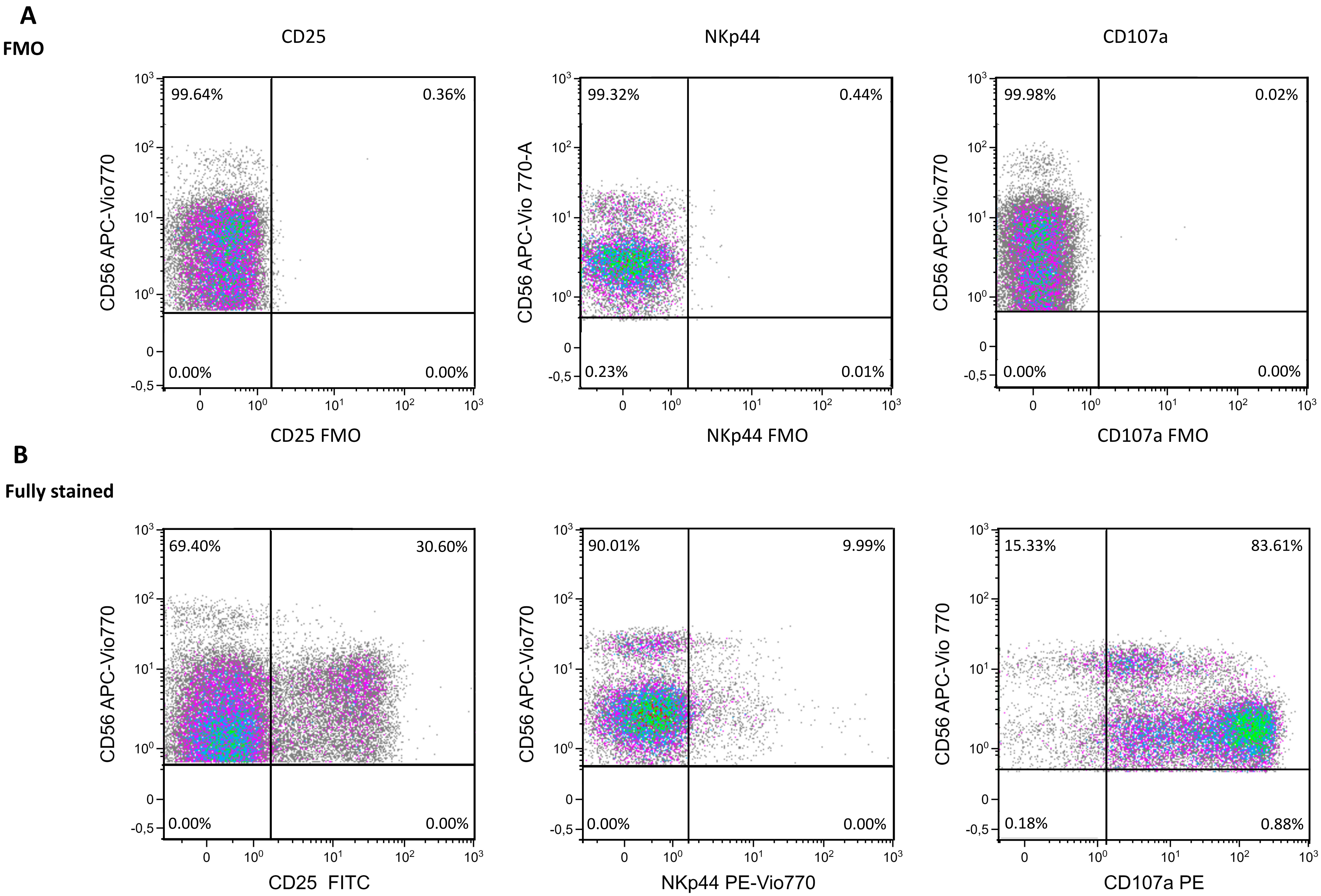

Supplementary figure 1: Fluorescence minus one (FMO) controls for gating NK function panel receptors

In case of NK cell function panel experiments, a Fluorescence Minus One (FMO) control was devised to optimize the identification of NK cell activation antigens CD25, NKp44 and CD107a under different stimulated conditions. FMO stains and a fully stained sample of NK cell stimulated with cetuximab coated A431 tumor target cells is shown in figure A and B. The FMO gate clearly indicates the negative control position.

Supplementary Fig. S2

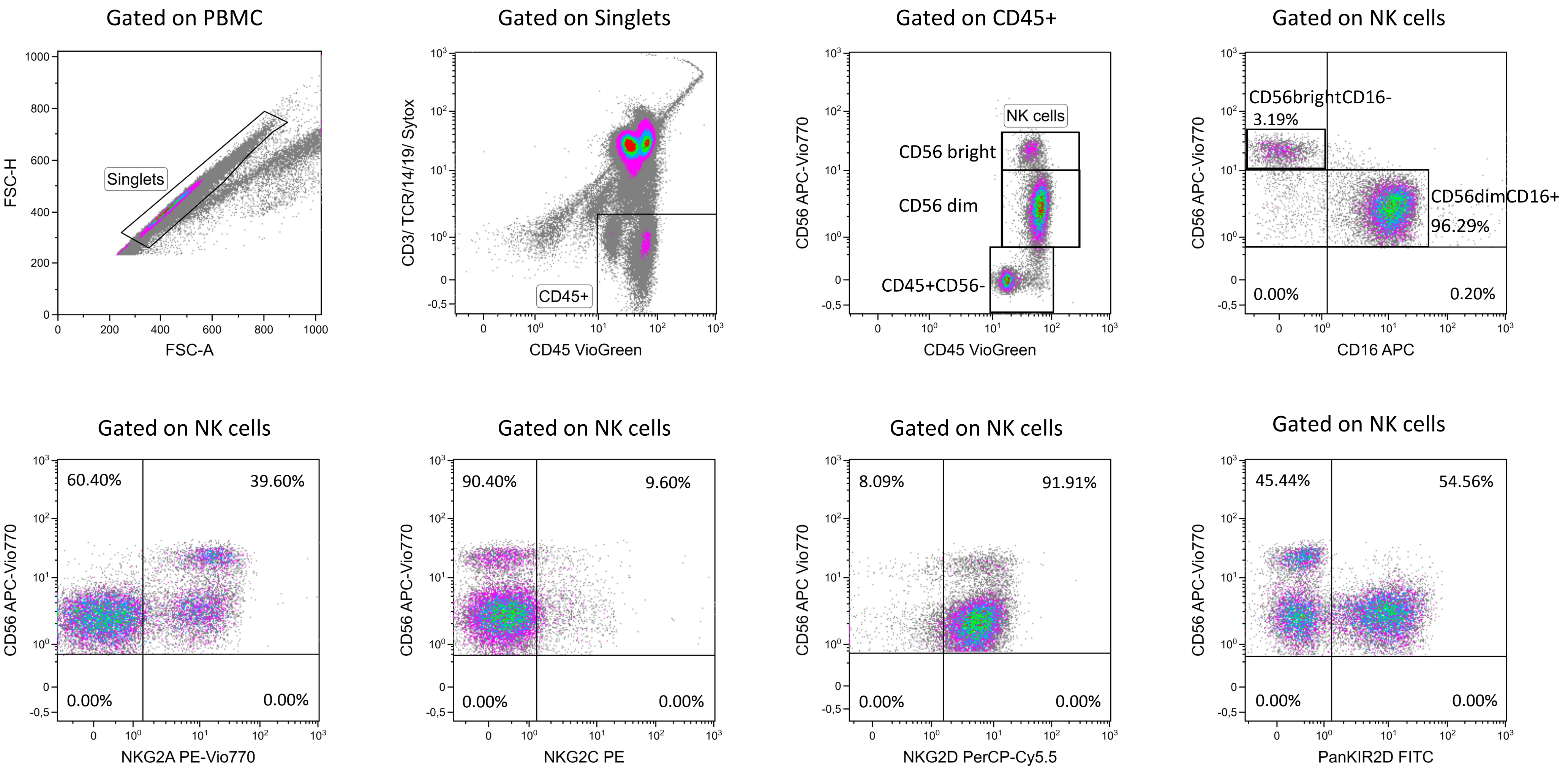

Supplementary figure 2: Gating strategy for the NK cell phenotype panel

The gating procedure to analyse NK cell phenotypes from PBMC population is shown. Cells acquired were gated FSC-H/FSC-A to eliminate doublets, followed by gating on the exclusion channel (CD3/TCRγδ/CD14/CD19/SYTOX® Blue) against CD45. Lymphocytes negative for exclusion channel antigens and positive for CD45 were then plotted for CD45+CD56+ cells. CD56+ NK cells were then gated to define single receptor positive cells CD56+CD16+, CD56+NKG2A+, CD56+NKG2C+, CD56+NKG2D+, CD56+KIR2D+ plots from CD56+NK cells. Further to facilitate NK subset analysis, NK cells were sub-divided into NK bright and NK dim populations based on CD56 expression and CD16 levels. CD45+CD56- cells can also be studied using this gating strategy.

Supplementary Fig. S3

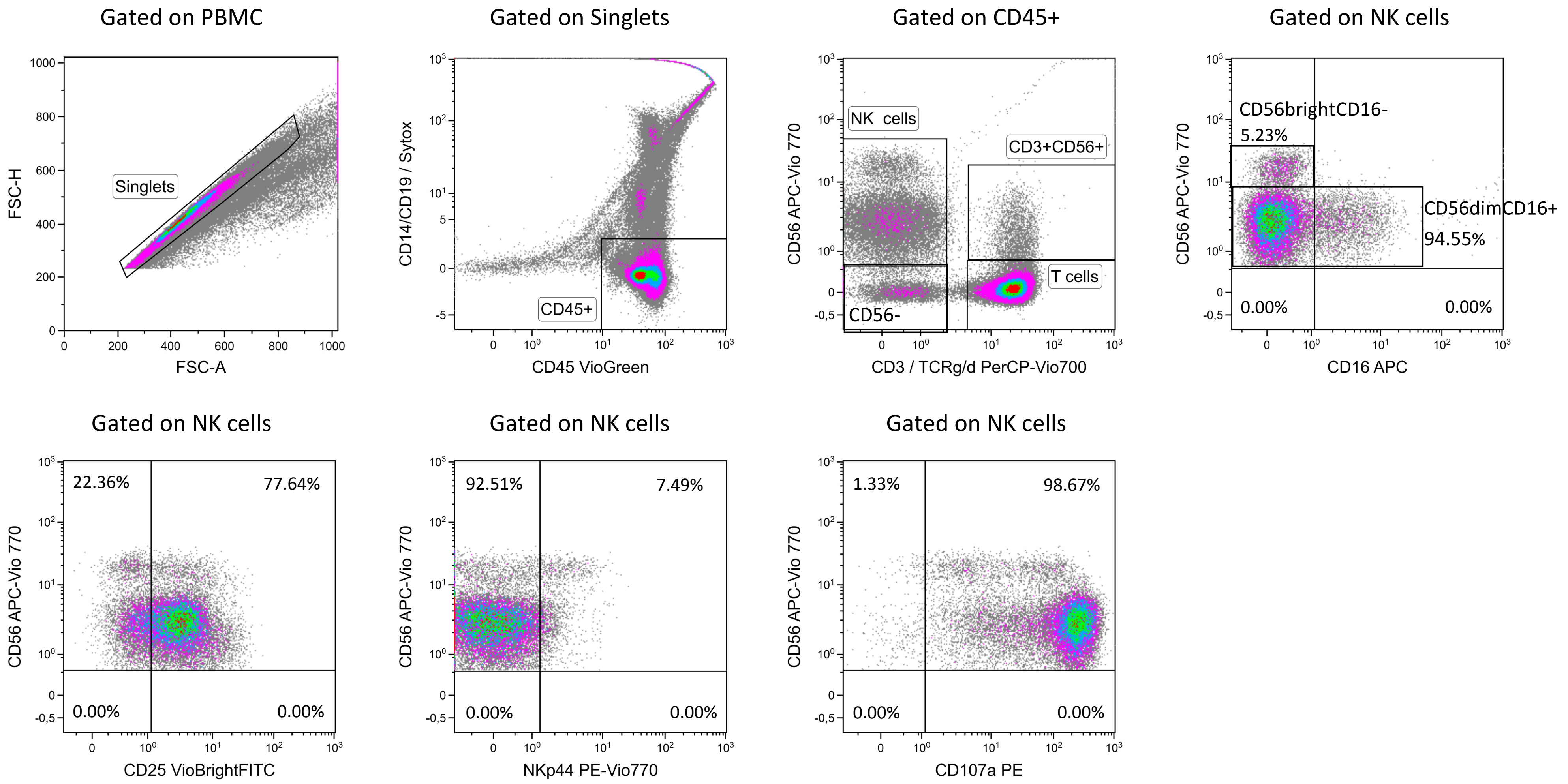

Supplementary figure 3: Gating strategy for the NK cell function panel

The gating procedure to analyse NK cell functions from PBMC population is shown. Singlets were selected gating PBMC against FCS-A versus FSC-H. CD45+ cells negative for CD14, CD19 and SYTOX® Blue were further gated against CD3/TCRγδ and CD56 to differentiate NK cells, T cells and CD3+CD56+ cells. CD45+CD56+ NK cells were further analysed for CD16, CD107a, NKp44 and CD25 expression. NK cells shown in this figure were stimulated with A431 + CET to show higher CD107a and lower CD16 levels. Further to facilitate NK subset analysis, NK cells were sub-divided into NK bright and NK dim populations based on CD56 expression and CD16 levels. CD45+CD56- cells can also be studied using this gating strategy.

Supplementary Fig. S4

Comparison of non-activated fresh versus cryopreserved NK cells

NK phenotype panel

(n=12)

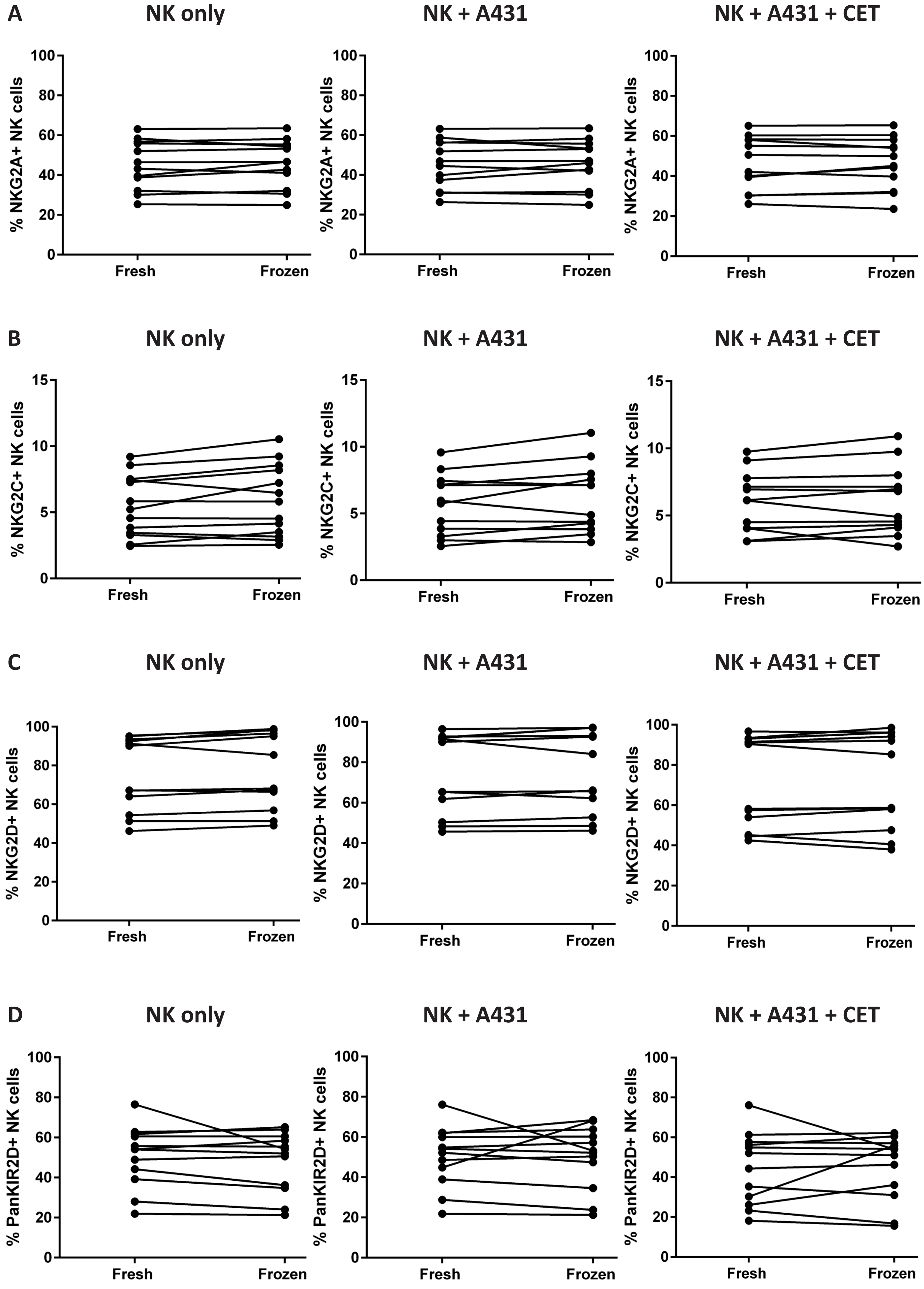

Supplementary figure 4: Comparison of NK phenotype panel marker differences between fresh and cryopreserved non-activated NK cells

Non-activated NK cells were stimulated with target cells (A431) alone or targets coated with cetuximab (CET). Expression levels of NKG2A (A), NKG2C (B), NKG2D (C), KIR2D (D) were compared between fresh and cryopreserved NK cells for the following conditions: i) NK only ii) NK + A431 and iii) NK + A431 + CET. Data points represent the mean of triplicate values from independent experiments from 12 PBMC donors (6 donors: BD LSRFortessa + 6 donors: MACSQuant). Statistical analysis was done using Wilcoxon test and no statistically significant differences were observed between non-activated fresh and cryopreserved NK cells.

Supplementary Fig. S5

Comparison of non-activated fresh versus cryopreserved NK cells

NK function panel

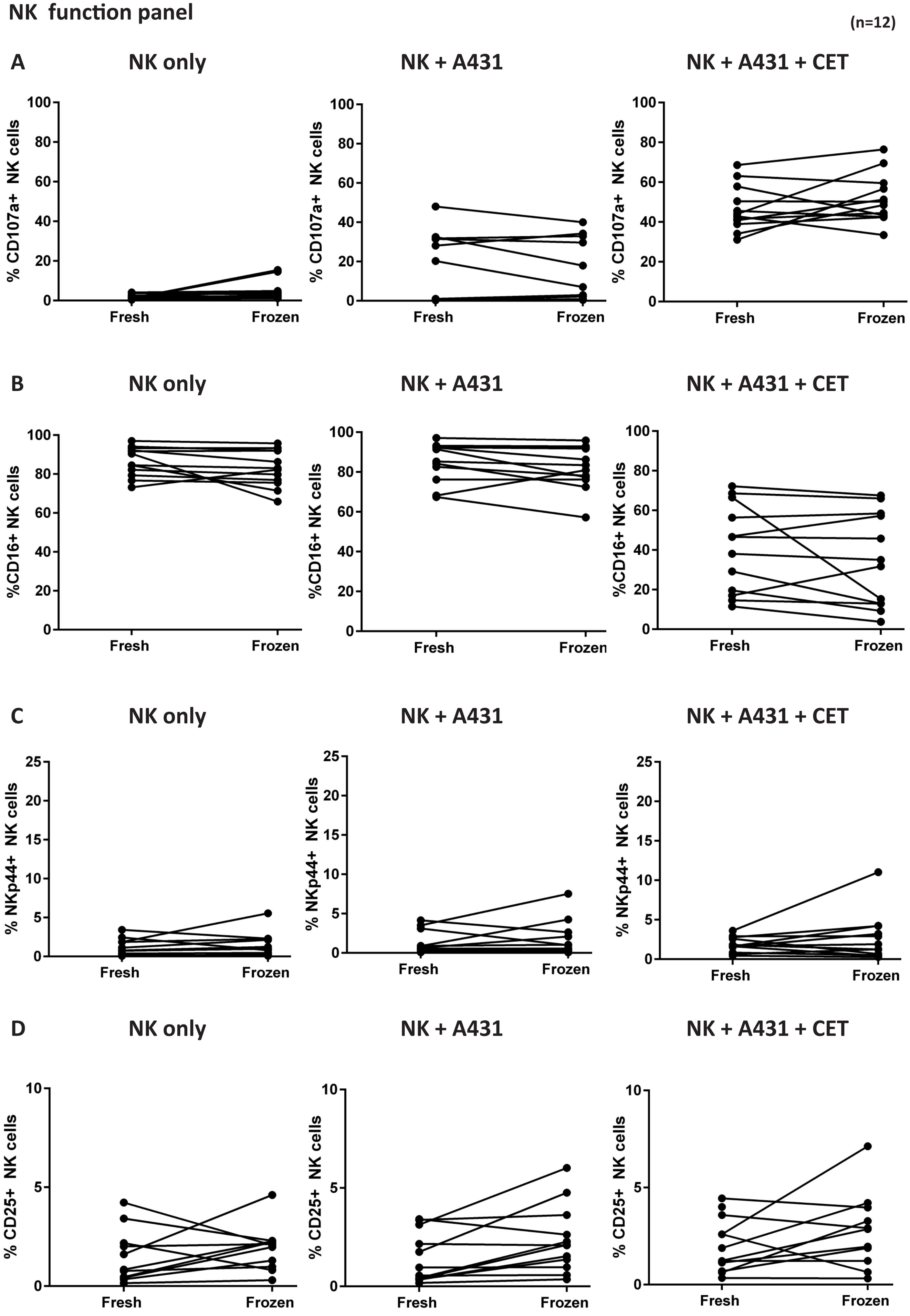

Supplementary figure 5: Comparison of NK function panel marker differences between fresh and cryopreserved non-activated NK cells

The capacity of NK cells to perform natural cytotoxicity and ADCC under different NK stimulated conditions were analysed on 12 healthy donors using the NK function panel antibody mix. Expression levels of CD107a (A), CD16 (B) NKp44 (C) and CD25 (D) were compared between fresh and cryopreserved non-activated NK cells for the following conditions: i) NK only ii) NK + A431 and iii) NK + A431 + CET. Data points represent the mean of triplicate values from independent experiments from 12 PBMC donors (6 donors: BD LSRFortessa + 6 donors: MACSQuant). Statistical analysis was done using Wilcoxon test and no statistically significant differences were observed between fresh and cryopreserved non-activated NK cells.

Supplementary Fig. S6

Comparison of cytokine activated fresh versus cryopreserved NK cells

NK phenotype panel

(n=12)

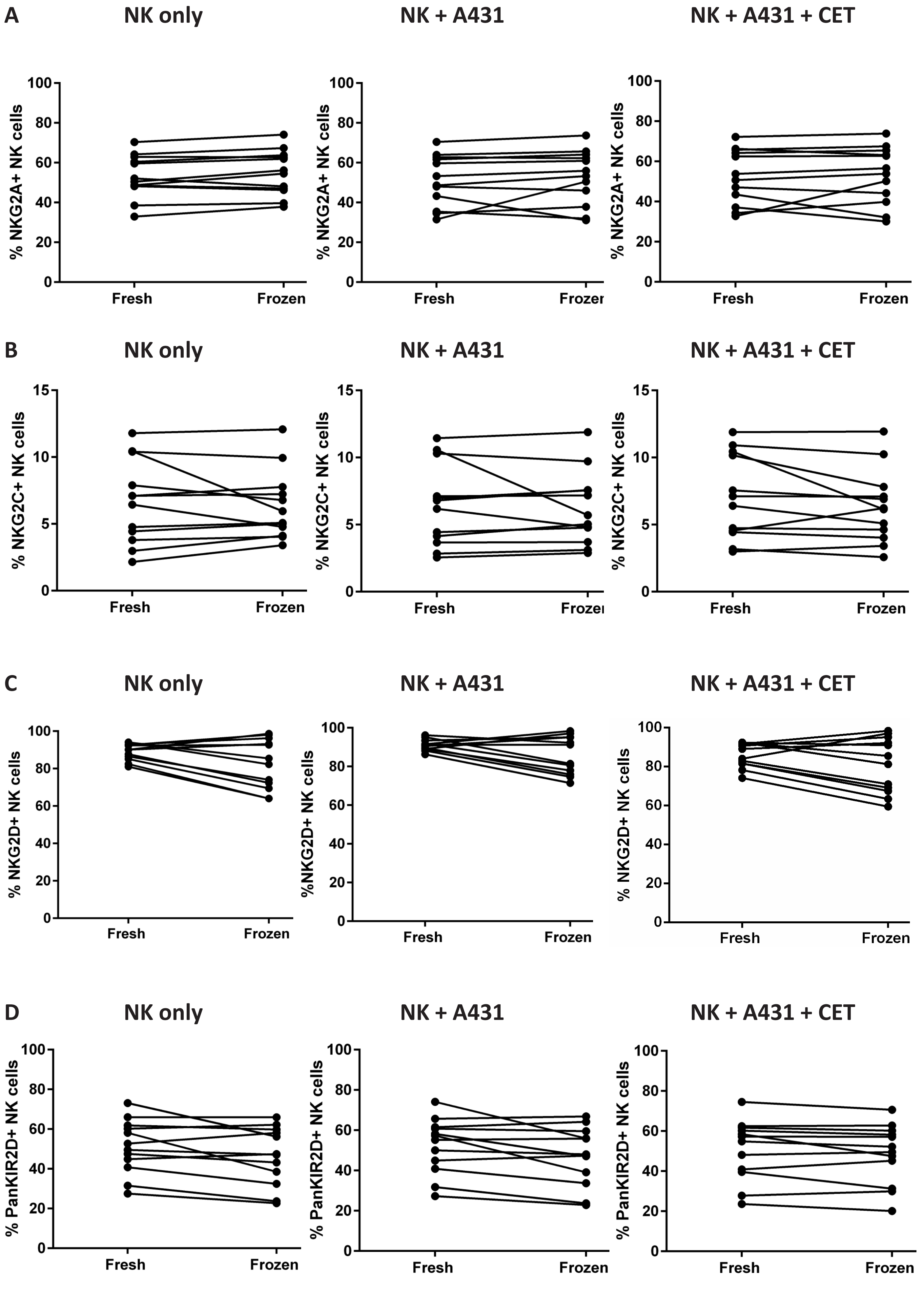

Supplementary figure 6: Comparison of NK phenotype panel marker differences between fresh and cryopreserved cytokine activated NK cells

NK cells were activated overnight with IL-2 and IL-15 and stimulated with target cells (A431) alone or targets coated with cetuximab (CET). Expression levels of NKG2A (A), NKG2C (B), NKG2D (C), KIR2D (D) were compared between fresh and cryopreserved NK cells for the following conditions: i) NK only ii) NK + A431 and iii) NK + A431 + CET. Data points represent the mean of triplicate values from independent experiments from 12 PBMC donors (6 donors: BD LSRFortessa + 6 donors: MACSQuant). Statistical analysis was done using Wilcoxon test and no statistically significant differences were observed between fresh and cryopreserved cytokine activated NK cells.

Supplementary Fig. S7

Comparison of cytokine activated fresh versus cryopreserved NK cells

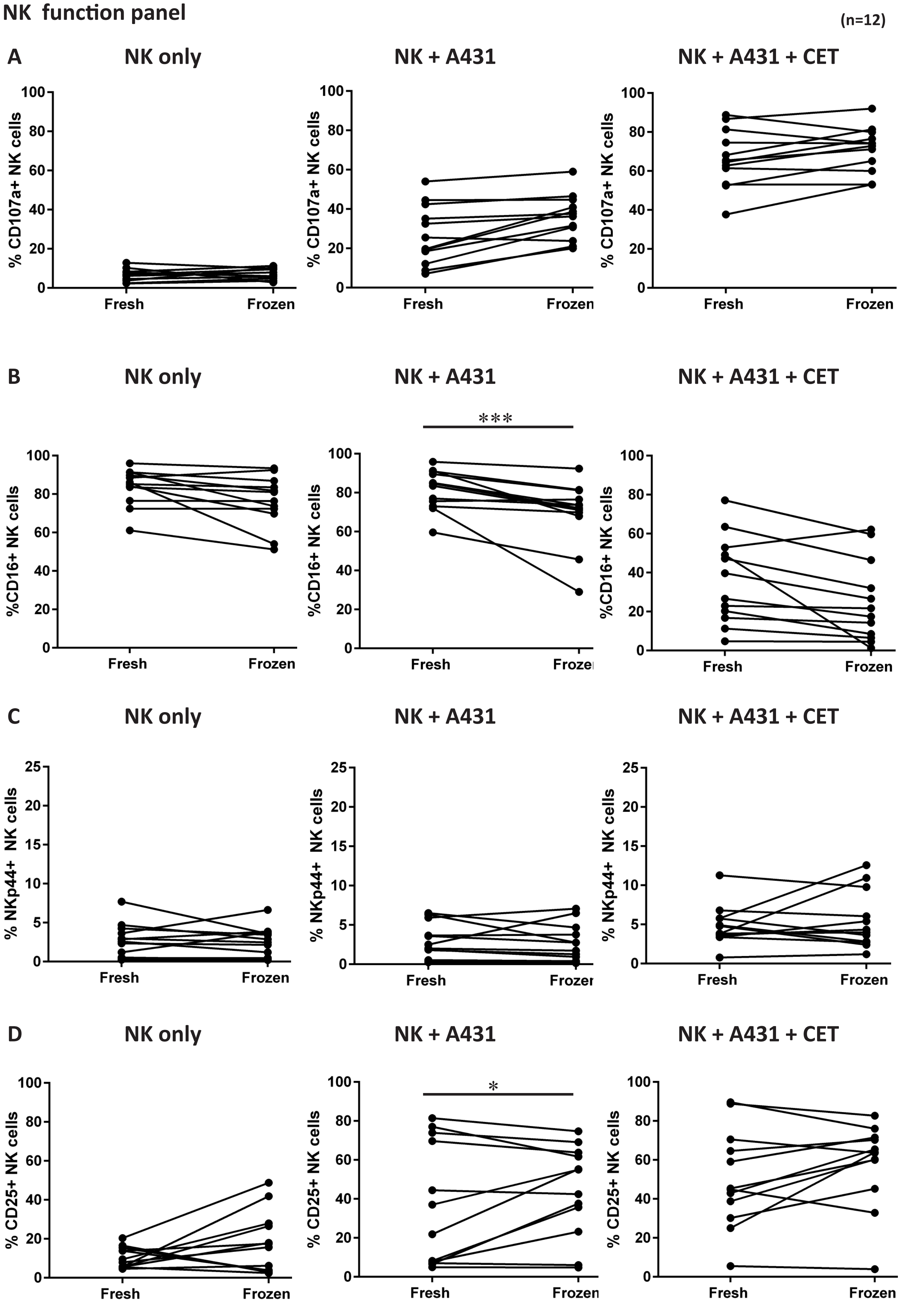

Supplementary figure 7: Comparison of NK function panel marker differences between fresh and cryopreserved cytokine activated NK cells

The capacity of NK cells to perform natural cytotoxicity and ADCC under different NK stimulated conditions were analysed on 12 healthy donors using the NK function panel antibody mix. Expression levels of CD107a (A), CD16 (B), NKp44 and CD25 (D) were compared between fresh and cryopreserved cytokine activated NK cells for the following conditions: i) NK only ii) NK + A431 and iii) NK + A431 + CET . Data points represent the mean of triplicate values from independent experiments from 12 PBMC donors (6 donors: BD LSRFortessa + 6 donors: MACSQuant). Statistical analysis was done using Wilcoxon test and only statistically significant differences are mentioned (*$p<0.05$, ***$p<0.001$).

Supplementary Fig. S8

Modified gating strategy to gate on CD56dimCD16+ NK cells

Gated on Singlets **Gated on CD45+ cells** **Gated on NK cells**

A

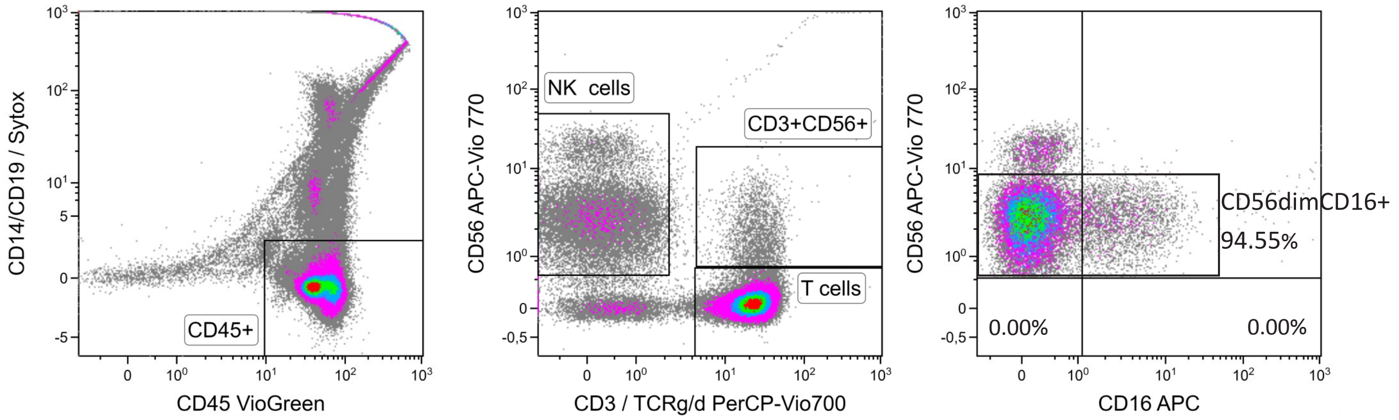

(n=12)

Comparison of non-activated fresh versus cryopreserved CD56dimCD16+ NK cells

B **NK only** **NK + A431** **NK + A431 + CET**

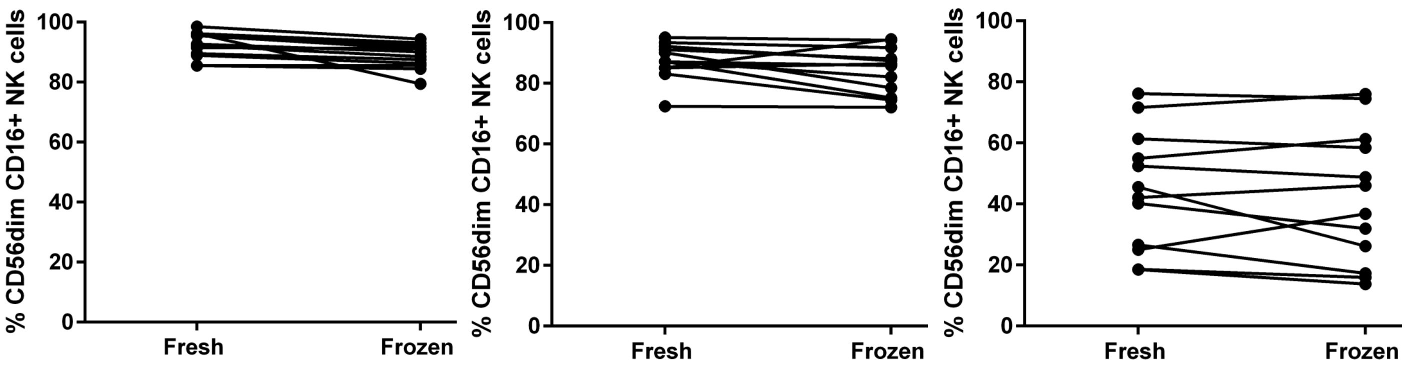

Comparison of cytokine activated fresh versus cryopreserved CD56dimCD16+ NK cells

C **NK only** **NK + A431** **NK + A431 + CET**

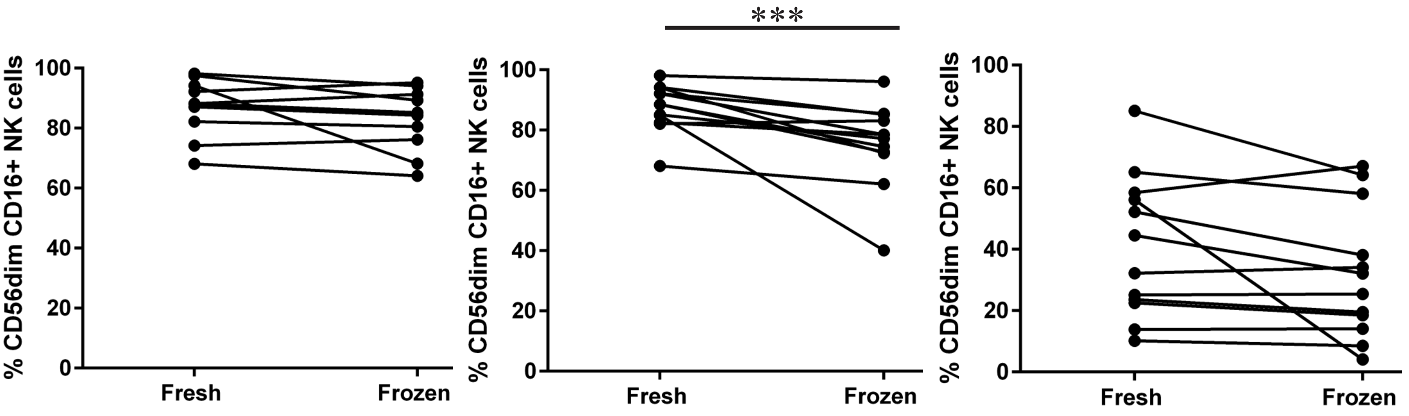

Supplementary figure 8: Paired comparison analysis of CD56dim CD16+ subset using modifed NK cell gating strategy

(A) The gating procedure to analyse NK cell phenotypes from PBMC population is shown. Lymphocytes negative for exclusion channel antigens and positive for CD45 were plotted for CD45+CD56+ cells. CD56+ NK cells were sub-divided into NK bright and NK dim populations based on CD56 expression and further the CD56dimCD16+ NK cells (NK effectors) were defined by gating CD45+CD3-CD56+ NK cells against CD16. (B) CD16 levels were compared between non activated fresh and cryo-preserved NK cells (C) CD16 levels were compared between cytokine activated fresh and cryopreserved NK cells. In, B and C the following conditions: i) NK only ii) NK + A431 and iii) NK + A431 + CET were compared between fresh and cryo-preserved NK cells for changes in CD56dim CD16+ marker expression. Data points represent the mean of triplicate values from independent experiments from 12 PBMC donors (6 donors: BD LSRFortessa + 6 donors: MACSQuant). Statistical analysis was done using Wilcoxon test and only statistically significant differences are mentioned (***$p<0.001$).

Supplementary Table S1

Compensation settings for BD FACSCanto™ II

Compensation matrix - NK phenotype panel BD FACSCanto™ II								
	VioGreen	VioBlue	FITC	PE	PerCP-Vio700	PE -Vio770	APC	APC-Vio770
VioGreen		4.52	0.00	0.00	0.00	0.00	0.00	0.00
VioBlue	2.42		0.00	0.00	0.00	0.00	0.00	0.00
FITC	0.00	0.00		7.45	0.00	0.00	0.00	0.00
PE	0.00	0.00	6.15		0.00	0.12	0.00	0.00
PerCP-Vio700	0.00	0.00	0.09	0.75		0.32	0.75	0.00
PE-Vio770	0.00	0.00	0.00	0.02	1.45		0.00	0.00
APC	0.00	0.00	0.00	0.00	0.10	0.00		4.52
APC-Vio770	0.00	0.00	0.00	0.00	0.00	0.00	1.55	

Compensation matrix - NK function panel BD FACSCanto™ II								
	VioGreen	VioBlue	FITC	PE	PerCP-Vio700	PE -Vio770	APC	APC-Vio770
VioGreen		7.52	0.00	1.53	0.00	0.00	0.00	0.00
VioBlue	9.50		0.00	0.00	0.00	0.00	0.00	0.00
FITC	0.00	0.00		9.52	0.00	0.00	0.00	0.00
PE	0.00	0.00	11.53		0.00	5.21	0.00	0.00
PerCP-Vio700	0.00	0.00	0.84	1.46		0.00	3.21	0.54
PE-Vio770	0.00	0.00	0.00	0.75	2.54		2.46	1.85
APC	0.00	0.00	0.00	0.00	0.00	0.00		6.53
APC-Vio770	0.00	0.00	0.00	0.00	0.00	0.00	4.85	

Supplementary Table S2

Compensation settings for BD LSRFortessa™

Compensation matrix - NK phenotype panel BD LSRFortessa™								
	VioGreen	VioBlue	FITC	PE	PerCP-Vio700	PE -Vio770	APC	APC-Vio770
VioGreen		7.54	0.00	0.00	0.00	0.00	0.00	0.00
VioBlue	8.41		0.00	0.00	0.00	0.00	0.00	0.00
FITC	0.00	0.00		8.19	0.00	0.42	0.00	0.00
PE	0.00	0.00	17.45		0.00	1.46	0.00	0.00
PerCP-Vio700	0.00	0.00	3.45	2.15		0.48	0.00	0.00
PE-Vio770	0.00	0.00	0.00	1.46	2.48		0.00	0.00
APC	0.00	0.00	0.00	0.00	1.45	0.00		10.25
APC-Vio770	0.00	0.00	0.00	0.00	0.45	0.00	4.51	

Compensation matrix - NK function panel BD LSRFortessa™								
	VioGreen	VioBlue	FITC	PE	PerCP-Vio700	PE -Vio770	APC	APC-Vio770
VioGreen		12.54	0.00	0.00	0.00	0.00	0.00	0.00
VioBlue	14.52		0.00	0.00	0.00	0.00	0.00	0.00
FITC	0.00	0.00		8.19	0.00	0.00	0.00	0.00
PE	0.00	0.00	11.89		0.00	5.47	0.00	0.00
PerCP-Vio700	0.00	0.00	7.92	6.55		0.47	0.00	0.00
PE-Vio770	0.00	0.00	0.00	4.18	5.21		0.00	0.00
APC	0.00	0.00	0.00	0.00	0.00	0.00		18.51
APC-Vio770	0.00	0.00	0.00	0.00	0.00	0.00	14.521	

Supplementary Table S3

Compensation settings for MACSQuant® Analyzer X

Compensation matrix - NK phenotype panel MACSQuant® Analyzer X

	VioGreen	VioBlue	FITC	PE	PerCP-Vio700	PE -Vio770	APC	APC-Vio770
VioGreen		0.95	0.00	0.00	0.00	0.00	0.00	0.00
VioBlue	0.69		0.00	0.00	0.00	0.00	0.00	0.00
FITC	0.00	0.00		7.52	0.00	0.00	0.00	0.00
PE	0.00	0.00	4.91		0.00	0.00	0.00	0.00
PerCP-Vio700	0.00	0.00	0.00	4.71		2.54	0.00	0.00
PE-Vio770	0.00	0.00	0.00	3.51	6.24		0.00	0.00
APC	0.00	0.00	0.00	0.00	0.00	0.00		5.27
APC-Vio770	0.00	0.00	0.00	0.00	0.00	0.00	11.41	

Compensation matrix - NK function panel MACSQuant® Analyzer X

	VioGreen	VioBlue	FITC	PE	PerCP-Vio700	PE -Vio770	APC	APC-Vio770
VioGreen		9.52	0.00	0.00	0.00	0.00	0.00	0.00
VioBlue	2.45		0.00	0.00	0.00	0.00	0.00	0.00
FITC	0.00	0.00		5.27	0.00	0.00	0.00	0.00
PE	0.00	0.00	11.52		0.00	0.00	0.00	0.00
PerCP-Vio700	0.00	0.00	0.57	3.94		5.27	0.00	0.00
PE-Vio770	0.00	0.00	0.00	0.00	7.52		0.00	0.00
APC	0.00	0.00	0.00	0.00	0.00	0.00		6.49
APC-Vio770	0.00	0.00	0.00	0.00	0.00	0.00	4.52	
